# Pelvic lipomatosis—A silent intruder in the pelvis: Clinical insights from a case report and literature analysis

**DOI:** 10.1097/MD.0000000000044107

**Published:** 2025-08-29

**Authors:** Shengming Zhou, Shiqi Sun, Qi Huang, Jiazhong Sun

**Affiliations:** a Department of Endocrinology, The First People’s Hospital of Xiaogan, Xiaogan, China; b Department of Clinical Laboratory, The Sixth Hospital of Wuhan, Affiliated Hospital of Jianghan University, Wuhan, China; c Department of Endocrinology, Zhongnan Hospital of Wuhan University, Wuhan, China.

**Keywords:** adipose tissue proliferation, imaging, obstructive symptoms, pelvic lipomatosis, pelvic mass, surgical management

## Abstract

**Rationale:**

This study aims to highlight the diagnostic challenges and multidisciplinary management of pelvic lipomatosis (PL), emphasizing imaging’s pivotal role and the need for early intervention to mitigate long-term morbidity. With fewer than 200 reported cases, PL remains underrecognized; this case underscores its potential to mimic common gastrointestinal/urinary disorders, advocating for heightened clinical suspicion.

**Patient concerns:**

A 42-year-old male presented with a 2-day history of colicky abdominal pain under the xiphoid process, ac companied by nausea, vomiting, and watery stools. Initial external hospital evaluations suggested small bowel obstruction and mesenteric lymphadenitis. Past medical history included abnormal blood glucose levels. Physical examination revealed right upper abdominal tenderness and mild distension.

**Diagnoses:**

Imaging studies were pivotal. Emergency abdominal–pelvic computed tomography showed diffuse low-density fat accumulation compressing the bladder and rectum (“pelvic lucency sign”), thickened bladder walls, and enlarged mesenteric lymph nodes. Subsequent magnetic resonance imaging confirmed symmetrical pelvic fat deposition with bladder deformation and elevated bladder base. Laboratory tests revealed mildly elevated bilirubin, C-reactive protein, and triglycerides, but no significant urinary or metabolic abnormalities. Final diagnosis confirmed PL with concurrent prostatitis and pelvic inflammatory changes.

**Interventions:**

The patient received conservative management, including acid suppression, gastric protection, nutritional support, and symptomatic relief. Surgical intervention was deferred due to symptom improvement. Post-discharge recommendations included dietary control, weight management, and regular follow-up for monitoring disease progression.

**Outcomes:**

Clinical resolution: symptoms resolved with conservative management including acid suppression and nutritional support, with the patient discharged in stable condition. Imaging correlation: magnetic resonance imaging confirmed symmetrical pelvic fat deposition with bladder deformation, while computed tomography demonstrated the characteristic “pelvic lucency sign.” Long-term planning: regular monitoring was instituted for potential urinary obstruction and malignancy risk given established associations with chronic cystitis.

**Lessons:**

PL poses diagnostic challenges due to nonspecific symptoms and often mimics gastrointestinal or urinary disorders. Imaging remains critical for accurate diagnosis. While conservative management suffices in mild cases, surgical options should be considered for severe organ compression. This case underscores the importance of early recognition, multidisciplinary evaluation, and tailored management to mitigate long-term morbidity.

## 1. Introduction

Pelvic lipomatosis (PL) is a rare condition involving excessive noncancerous fat growth in the pelvic cavity, first described in the 1960s with fewer than 200 cases reported. It can compress pelvic organs like the bladder and rectum, causing urinary and bowel issues, or even kidney problems in severe cases.^[1]^ The cause is unknown, though obesity, hormonal imbalances, or chronic inflammation are suspected. It mainly affects middle-aged men, with an 18:1 male-to-female ratio. Diagnosis is challenging due to vague symptoms. Computed tomography (CT) and magnetic resonance imaging (MRI) scans are key, showing symmetrical fat deposits and displaced organs. Biopsy is rarely needed. Treatment varies: mild cases may only require monitoring, while severe cases may need surgery to remove fat or relieve pressure. This article provides a comprehensive overview of the condition, including its epidemiology, diagnosis, and management, to raise awareness and improve patient care.

## 2. Patients and clinical evaluation

**Figure 1.**
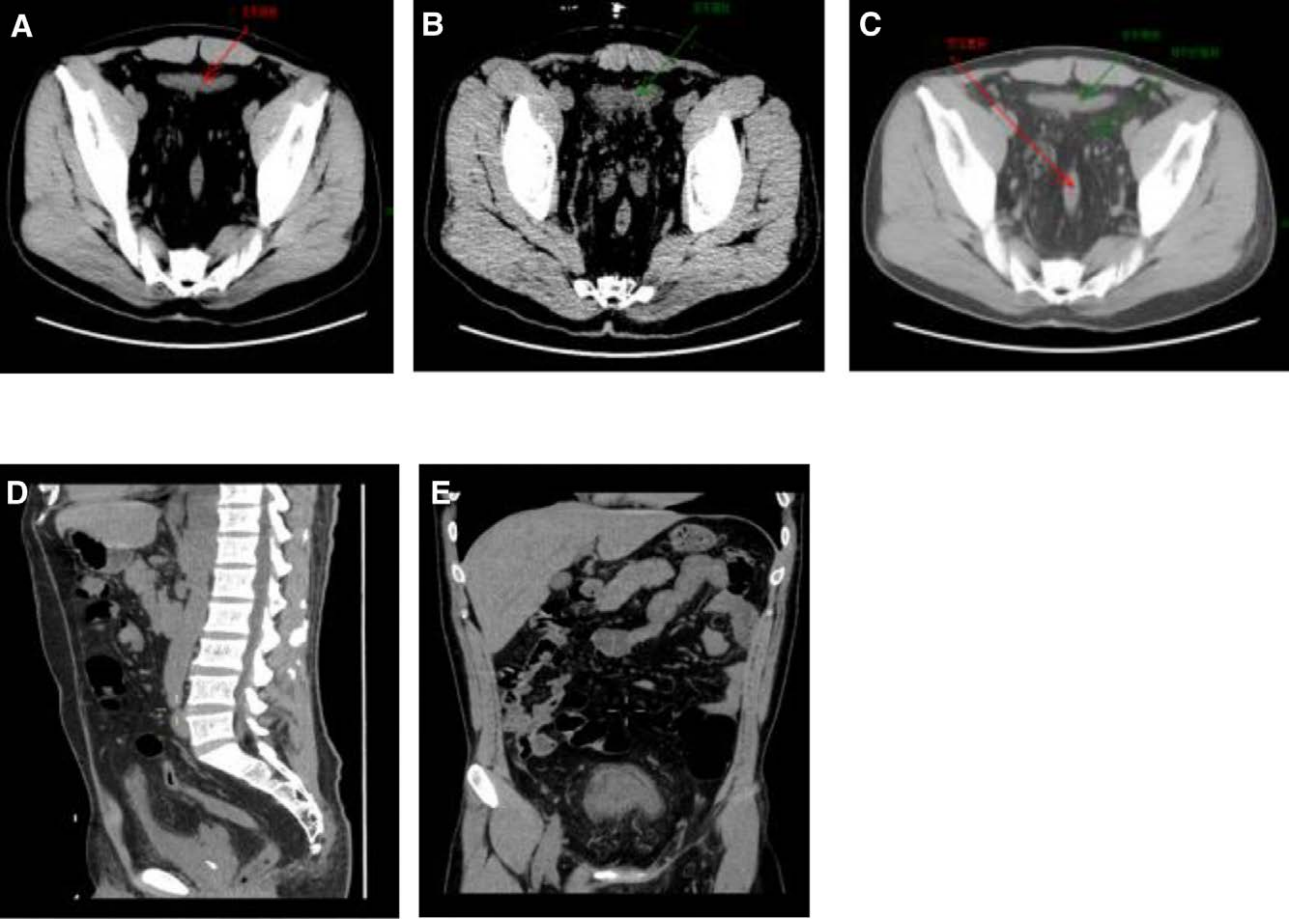
(A–E) Full abdomen + pelvic CT: (A–C) Horizontal CT scans show diffuse low-density fat accumulation around the bladder and rectum (pelvic transparency sign), with compression and deformation of the bladder and rectum, and poor bladder filling. The bladder wall is thickened and rough, and there are tortuous pelvic veins. (D, E) Sagittal and coronal CT scans show elevation and deformation of the bladder base in the sagittal view.

**Figure 2.**
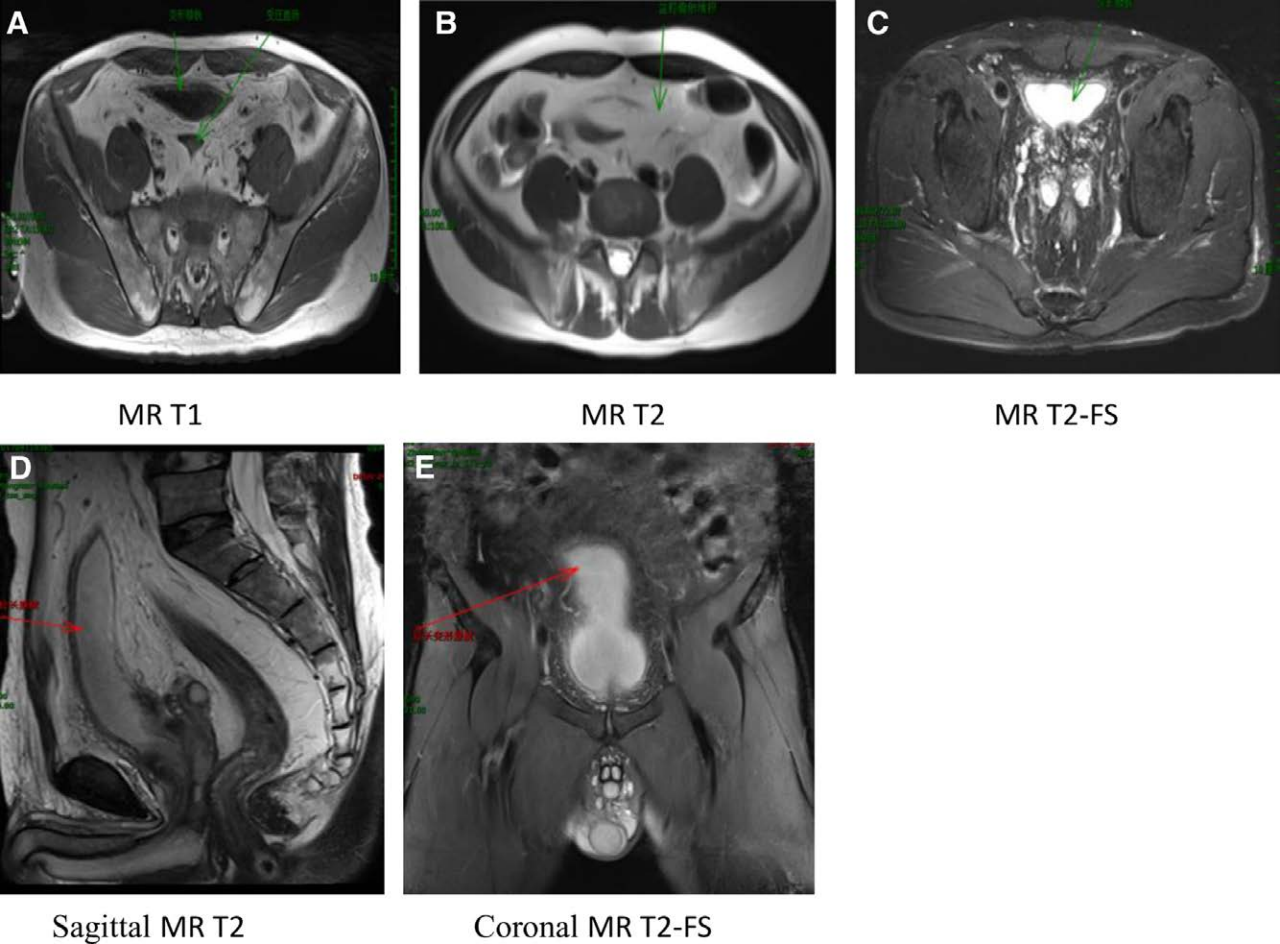
(A–E) Abdominopelvic MRI: (A–C) MRI shows a large amount of fat accumulation in the pelvic region, exhibiting short T1 and long T2 signals. The bladder and rectum are significantly compressed and deformed, with tortuous pelvic veins and notable changes in the position of the seminal vesicles. (D) The bladder base is visibly elevated, appearing long and slender like an eggplant or loofah, with deformation of the rectum, disappearance of the colonic tenia, and significant rigidity of the intestinal wall. (E) In the coronal fat-suppressed sequence, the deformed and inverted bladder, resembling a ping-pong paddle, can be observed. The bladder wall appears rough and thickened.

Patient, male, 42 years old, admitted to the hospital due to “abdominal pain accompanied by vomiting for 2 days.” Two days prior to admission, the patient experienced pain under the xiphoid process without obvious cause, characterized as colicky and persistent, without radiating pain, and not clearly related to eating. This was accompanied by nausea and vomiting, with the vomitus consisting of stomach contents. The abdominal pain relieved after vomiting. The patient had not defecated and did not experience chills, fever, heart palpitations, chest tightness, Negative for urinary urgency, frequency, dysuria, or other urethral symptoms, or other discomforts. He visited the emergency department of an external hospital, where he was diagnosed with “small bowel obstruction” and treated with gastrointestinal decompression, enema, and pain relief, which improved his symptoms. After the enema, he passed loose, watery stools. Seeking further treatment, he came to our hospital and was admitted to the gastroenterology department under the emergency diagnosis of “mesenteric lymphadenitis, acute gastroenteritis.” Past medical history: abnormal blood glucose; no other special medical history, and no special family history. Physical examination: T 36.5°C, HR 63 bpm, R 18 bpm, BP 134/79 mm Hg; height 178 cm, weight 70 kg, body mass index (BMI) 22.09 kg/m²; positive signs: tenderness in the right upper abdomen and under the xiphoid process, slightly distended abdomen, soft abdomen, no palpable masses, and no other special findings on the rest of the physical examination. Auxiliary examinations: external hospital liver, kidney, sugar, and electrolyte tests: blood potassium 3.17 mmol/L, with no other significant abnormalities. Coagulation profile, blood routine, and electrocardiogram showed no significant abnormalities. In our hospital’s emergency department, the urinalysis showed: Glucose (±) weakly positive; amylase and lipase levels were normal; blood routine tests and prostate-specific antigen were normal; electrocardiogram was normal. Hospital auxiliary examinations: Liver and kidney function, glucose, electrolytes, and C-reactive protein: Total bilirubin: 30.8 μmol/L↑; Indirect bilirubin: 26.5 μmol/L↑; Total protein: 60.4 g/L↓; Albumin: 39.0 g/L↓; Gamma-glutamyl transferase: 58 U/L↑; Glucose: 3.70 mmol/L↓; Calcium (Ca++): 1.97 mmol/L↓; High-sensitivity C-reactive protein: 5.68 mg/L↑; Ischemia-modified albumin: 53.3 KU/L↓; Serum amyloid A: 19.05 mg/L↑. Lipid profile: Triglycerides: 1.89 mmol/L↑; the rest were normal. Urinalysis: Protein: weakly positive (±); Urobilinogen: positive (+); White blood cells: weakly positive (±); White blood cells: 60.20/µL↑; Epithelial cells: 6.50/µL↑. Glycated hemoglobin test: Total glycated hemoglobin: 5.8%↓. Hepatitis B full set quantitative test: No abnormalities. Blood routine, coagulation profile, myocardial enzymes, and brain natriuretic peptide showed no significant abnormalities. Liver function panel: Total bilirubin: 32.3 μmol/L↑; Indirect bilirubin: 27.9 μmol/L↑; Gamma-glutamyl transferase: 60 U/L↑; the rest were normal. Imaging studies: Emergency CT of our hospital: Full abdomen + pelvic CT: Increased and enlarged mesenteric lymph nodes, mesenteric lymphadenitis? Soft tissue nodule at the bottom of the gallbladder, adenomyosis? Prostatitis, prostate calcification, pelvic inflammatory changes (see Fig. 1). Hospital colonoscopy: Colonic polyp EMR and titanium clip suture; Internal hemorrhoids. Gastroscopy: Reflux esophagitis (grade A); Chronic superficial gastritis. Hospital abdominal and pelvic MRI showed: Multiple slightly enlarged lymph nodes in the right mesocolon; abnormal bladder shape, possible cystitis; small liver cyst; small nodule at the bottom of the gallbladder, considered adenomyosis; possible pelvic lipomatosis; a small amount of abdominal fluid (see Fig. 2). The patient’s final discharge diagnosis: Pelvic lipomatosis. The patient was treated with acid suppression, stomach protection, nutritional support, and symptomatic treatment during hospitalization, and was discharged after symptoms improved. The patient was advised to control diet, weight, and to follow up regularly.

## 3. Discussion

PL is a rare benign proliferative disorder characterized by the unexplained overgrowth of pelvic adipose tissue, leading to compression and invasion of pelvic organs. Strictly speaking, it is a diffuse tumor of the retroperitoneal adipose tissue, but it is not malignant histologically. PL was first reported by Engels in 1959, who described the overgrowth of fat around the sigmoid colon and bladder, leading to fixation and deformation of these organs.^[2]^ Later, Fogg and Smyth formally named it “pelvic lipomatosis” in 1968, defining it as the excessive proliferation of normal adipose tissue in the pelvic space around the rectum and bladder.^[3]^ The proliferative adipose tissue envelops, compresses, and squeezes adjacent organs, causing deformation and displacement. This leads to a series of obstructive changes, such as obstruction of the lower ureter, bladder neck, posterior urethra, sigmoid colon, and rectum. These obstructive changes can result in hydronephrosis and ureteral dilation, ultimately leading to renal function decline and even uremia.

This disease is clinically rare, with fewer than 200 cases reported in the literature, primarily as individual case reports. In recent years, as awareness of the disease has increased, there has been a trend of an increasing number of cases reported in the literature. Previously, the reported incidence was low, with a rate of 0.6 to 1.7 per 100,000 people in the United States.^[4]^ The incidence is more common in males,^[5]^ with an age range of 20 to 62 years and an average age of 48 years. There are also reports of cases in females and children,^[6,7]^ and in rare instances, familial occurrences.^[8]^ There are racial and gender differences in the incidence; in the United States, the incidence in Black individuals is twice that of White individuals, with a male-to-female ratio of 18:1. However, the true incidence may be underestimated due to the difficulty in identifying the disease, which is often only discovered when late-stage complications arise.

The etiology of PL remains not entirely clear. Some literature suggests that it may be related to chronic pelvic inflammation caused by chronic urinary tract infections, hormonal metabolic disorders, congenital venous malformations in the pelvis, lipoprotein metabolism disorders, local allergic reactions, and endocrine disturbances. The relationship between obesity and PL is ambiguous, but it is not associated with diabetes.^[9]^ Morettin et al^[10]^ consider overweight to be a potential cause of PL. In contrast, Craig et al^[11]^ argue that obesity cannot explain the racial predisposition to PL. Literature shows that two-thirds of reported cases in the United States involve obese patients.^[12]^ However, this is not the case in Japan.^[13]^ On the other hand, it has been reported that PL can be influenced by both weight loss and weight gain.^[14]^ According to the World Health Organization (2004), the average BMI of PL patients is currently 25.5 ± 3.0, which does not significantly exceed the normal range (18.5–25.0). Additionally, there have been reports of a familial genetic tendency for the disease, and its genetic and pathogenic mechanisms may be related to mutations in the HMGI-C gene.^[15]^ Kume et al^[16]^ reported cases of achondroplasia complicated by pelvic lipomatosis, and since achondroplasia is a congenital genetic disorder, it is speculated that pelvic lipomatosis may be related to congenital or genetic factors. This case involves a young male patient, 178 cm tall, weighing 70 kg, with a BMI of 22.09 kg/m²; he is not an obese patient, has no family history of related diseases, a past history of abnormal blood sugar, weakly positive urine glucose, weakly positive urine white blood cells, and mildly abnormal lipid profiles.

The clinical manifestations of PL are often nonspecific, and early stages may present with no obvious symptoms. Despite its low incidence and benign nature, as pelvic fat continues to proliferate, the compression of the bladder and rectum increases, leading to corresponding symptoms. Nearly half of PL patients experience urinary symptoms due to obstructive nephropathy and associated chronic cystitis, including hematuria, bladder irritation symptoms, difficulty urinating, a sensation of incomplete bladder emptying, gross pyuria, and acute urinary retention. Approximately 40% of patients develop renal failure within an average of 5 years after diagnosis.^[17]^ Additionally, 75% of patients are accompanied by cystitis glandularis, cystic cystitis, and follicular cystitis.^[18]^ It is known that cystitis glandularis and cystic cystitis are considered potential precancerous lesions for bladder adenocarcinoma, and the association between cystitis glandularis and bladder adenocarcinoma has been documented in the literature.^[19]^ About 25% of patients present with constipation as the primary gastrointestinal symptom,^[1]^ while other digestive manifestations include nausea, vomiting, abdominal pain, diarrhea, hematochezia, and anorexia. Approximately 30% of patients have concurrent hypertension. Other symptoms may include lower back pain, pelvic discomfort, lower limb edema, and upper abdominal fullness. In this case, imaging revealed prostatic and bladder inflammation, but the patient had no obvious urinary symptoms or a history of hypertension. The primary symptoms were abdominal pain and vomiting, which are gastrointestinal-related, making it easy to misdiagnose as a digestive disorder.

Due to the nonspecific clinical manifestations of PL, the characteristic imaging features of pelvic lipomatosis serve as the primary basis for diagnosis. Therefore, imaging plays a crucial role in the diagnosis of this condition. Various imaging methods have been reported in the literature, including X-ray cystography, excretory urography, barium enema, ultrasound, CT, and MRI. Moss et al considered X-ray cystography, intravenous pyelography, and barium enema findings as indirect signs, characterized by elongation and deformation of the bladder neck, elevation of the bladder base, medial displacement of the lower ureters, and straightening of the sigmoid colon due to compression. IVP may reveal a characteristic “pear-shaped,” “teardrop-shaped,” or “gourd-shaped” bladder with external compression and elongation, as well as elevation of the bladder base. Additionally, signs of upper urinary tract hydronephrosis may be observed.^[20]^ Ultrasound is less ideal for imaging the lower abdomen, but some reports suggest that combining pelvic ultrasound with CT can improve detection rates.^[21]^ CT is effective in distinguishing fat from other tissues and can provide a qualitative diagnosis, making it the most effective and fundamental imaging modality.^[22]^ CT may show characteristic changes, such as the “pelvic lucency sign,”^[23]^ which reflects the presence of abundant fat in the pelvis. This is seen as a large area of uniform low-density shadow around the bladder and rectum, with CT values around ‐100 Hounsfield units and no significant enhancement. The rectal lumen may be compressed and deformed, and sagittal views may show abnormal bladder morphology with elevation of the bladder base, which are characteristic features of this condition. MRI offers high tissue resolution, and fat tissue exhibits characteristic signal patterns on MRI, appearing as high signal intensity on both T1-weighted and T2-weighted images. Fat suppression sequences can selectively suppress fat signals, turning them into low signal intensity. Additionally, MRI allows for multi-planar imaging from various angles, providing a clear view of the deformed bladder, rectal compression, and the extent of surrounding fat tissue thickening. Magnetic resonance urography can also provide a three-dimensional visualization of the compressed bladder, tortuous and dilated ureters, and the narrowed segment at the ureterovesical junction. Therefore, MRI has become the preferred method for diagnosing this condition.^[24]^ The imaging findings in this case are consistent with the characteristic features of PL, confirming the diagnosis.

Due to the unclear etiology, there is no definitive treatment strategy for PL, and management is primarily symptom-based. In cases of obesity, weight loss can have a positive impact on the disease. Conservative treatments, such as weight loss, long-term antibiotic therapy, hormonal therapy, and radiation therapy, have shown limited efficacy but may yield varying results. If obstruction develops, surgical intervention may be necessary, including pelvic fat removal, ureteral lysis, ureteroneocystostomy, bladder neck resection, nephrostomy, or ureterostomy.^[25]^ In a study by Klein et al,^[17]^ 39% of patients required definitive urinary diversion within 7 years. Some literature suggests that pelvic lipectomy is not advisable due to the adhesion of important structures to the abnormal fat and the lack of clear anatomical planes. However, there are also reports of successful surgical cases involving the removal of pelvic fat tissue.^[26]^ Ali et al^[27]^ successfully performed bladder-preserving resection of pelvic lipomatosis using the bladder wall as an anatomical guide, avoiding urinary diversion. They recommended surgical resection of pelvic fat with bladder preservation as a treatment option for PL patients with persistent pelvic pain and voiding dysfunction. However, there are also cases of recurrence or surgical failure. Therefore, the choice of surgical intervention remains controversial.

Carpenter et al^[26]^ proposed categorizing patients into 2 groups. The first group consists of young, stocky males under 50 years old with significant lower urinary tract symptoms, pelvic discomfort, and hypertension. These patients are at risk of developing ureteral obstruction and uremia. The second group includes patients over 50 years’ old who are incidentally diagnosed during evaluations for unrelated issues, with mild symptoms and a low risk of disease progression. Klein et al^[17]^ suggested that the first group, often robust with bladder morphological changes and irritative symptoms, tends to progress rapidly, leading to urinary obstruction or uremia, and thus requires earlier surgical intervention. The second group, with slower disease progression, may remain stable for 10 years or longer and should be monitored regularly. This includes renal function tests every 6 months, imaging every 2 years, and periodic cystoscopy, with surgery considered if necessary. Cystoscopy and biopsy have shown proliferative cystitis in 75% of cases and cystitis glandularis in 40% of cases. Annual cystoscopy is recommended for long-term monitoring of potential bladder adenocarcinoma risk.

The patient demonstrated favorable clinical resolution with conservative management (acid suppression therapy and nutritional support), remaining symptom-free at 3-month follow-up. Imaging findings were diagnostic, with MRI revealing symmetrical fat deposition causing bladder deformation (Fig. 2) and CT showing pathognomonic “pelvic lucency sign” (Fig. 1). Given the known association between PL and chronic cystitis-related complications, long-term surveillance protocols were emphasized, including: annual cystoscopy to monitor for cystitis glandularis progression; biannual renal function assessments; and periodic imaging to evaluate for urinary tract obstruction. This comprehensive approach addresses both immediate symptom management and potential malignancy risks associated with chronic bladder inflammation.

In this case, the patient is a young, robust male in the first group but lacks significant clinical symptoms. Imaging revealed prostatic and bladder inflammation, though the single-center case report design limits generalizability of these findings. Notably, cystoscopy was not performed, which may have missed concurrent cystitis glandularis and limited the comprehensive evaluation of bladder pathology. The patient was advised to lose weight and undergo regular follow-up. Given the unavailability of long-term follow-up data, progression risks including potential malignant transformation could not be fully characterized. Future clinical evaluations of this patient will incorporate cystoscopy and urine cytology to provide more comprehensive diagnostic information, as these procedures are essential for complete urological assessment.

## Author contributions

**Conceptualization:** Jiazhong Sun.

**Data curation:** Shengming Zhou, Shiqi Sun, Qi Huang, Jiazhong Sun.

**Formal analysis:** Qi Huang.

**Funding acquisition:** Jiazhong Sun.

**Investigation:** Shiqi Sun.

**Methodology:** Shengming Zhou, Shiqi Sun, Qi Huang, Jiazhong Sun.

**Resources:** Shengming Zhou, Qi Huang.

**Supervision:** Jiazhong Sun.

**Validation:** Shengming Zhou, Shiqi Sun.

**Visualization:** Shengming Zhou, Jiazhong Sun.

**Writing – original draft:** Shengming Zhou, Shiqi Sun, Jiazhong Sun.
